# The Influence of In-Mould Annealing and Accelerated Ageing on the Properties of Impact-Modified Poly(Lactic Acid)/Biochar Composites

**DOI:** 10.3390/polym16223102

**Published:** 2024-11-05

**Authors:** Pavel Brdlík, Jan Novák, Martin Borůvka, Jaume Gomez-Caturla, Petr Lenfeld

**Affiliations:** 1 Faculty of Mechanical Engineering, Technical University of Liberec, Studentská 1402/2, 46117 Liberec, Czech Republic; jan.novak@tul.cz (J.N.); martin.boruvka@tul.cz (M.B.); petr.lenfeld@tul.cz (P.L.); 2 Institute of Materials Technology (ITM), Universitat Politecnica de Valencia (UPV), Plaza Ferrandiz y Carbonell 1, 03801 Alcoy, Spain; jaugoca@epsa.upv.es

**Keywords:** PLA, biochar, ageing, particle size, impact modifier, mechanical properties, mould temperature

## Abstract

In the last few decades, a large number of natural additives have been analysed in connection with the improvement of the properties of poly(lactic acid) (PLA) bioplastic materials. This article comprehensively analyses the applicability of a highly stable and progressive multifunctional additive produced from renewable resources—biochar. The effect of biochar on the structural development and various thermo-mechanical properties was evaluated as a function of the biochar size and volume, addition of an impact modifier and in-mould annealing during injection moulding. In addition, the effect of accelerated ageing on the change in properties was also analysed. The evaluated results showed a significant influence of the particle size and biochar content on the properties of PLA biocomposites. However, the crucial aspect was the production process with a higher mould temperature and longer production time. Consequently, the effect of additives with adjusted processing worked synergistically on the performance of the resulting biocomposites. The accelerated ageing process did not induce any significant changes in the mechanical, impact and heat resistance behaviour of neat PLA. On the other hand, significant effects on the behaviour of the modified PLA biocomposites were observed. Impact-modified PLA achieved a toughness of 28 kJ/m^2^, an increase of 61% compared to neat PLA. Similar observations were made when submicron biochar was incorporated into the PLA matrix (a 22% increase with PLA/5B1). These increases were even more pronounced when injected into a 100 °C mould. Due to the synergistic effect, excellent impact toughness results of 95 kJ/m^2^ (a 428% increase) were achieved with PLA/IM/5B1. Moreover, these results persisted even after accelerated ageing.

## 1. Introduction

Due to the increasing pollution of the planet and the depletion of raw materials, bio-based and biodegradable plastics are one of the most discussed topics today. In the last decade, poly(lactic acid) (PLA) has become one of the most commercially used renewable materials. Unfortunately, its applicability is limited by certain constraints. These include low heat resistance and high brittleness. There are several modifications that can overcome these limitations. The most commonly used modification of PLA’s properties is the incorporation of additives. Hence, several types of additives produced from renewable sources, such as plasticisers [1,2,3,4,5,6], nucleating agents [7,8,9,10,11,12], natural fibres [13,14,15,16,17,18,19,20] and fibres, have been analysed in the last decade. The problem with many of these additives is the process and functional stability [21,22,23]. PLA is considered to be a biodegradable material under industrial composting conditions. Our previous results revealed its high degradation kinetics only in thermophilic environments [24,25]. The mesophilic conditions of compost (home composting) and water environment release larger fragments that are not easily assimilated by microorganisms. This led to the low biodegradation kinetics of PLA and the generation of microplastics, which can be a significant environmental problem due to their ability to absorb toxic pollutants (heavy metals, fertilisers, etc.) [26,27]. Therefore, a vital aspect is to find suitable additives to improve the limiting properties of PLA and the biodegradation kinetics at the same time.

One of the most promising materials addressing the above requirements is biochar. Biochar is a carbon-rich material characterised by high stability (chemical and thermal), high electrical conductivity [28], high specific surface area [29,30], and ability to immobilise contaminants (heavy metals) [31,32,33]. Biochar applied as a particulate filler can perform several functions simultaneously. It can be used as a heterogeneous nucleating agent for semi-crystalline plastics, as a colorant, as a UV stabiliser, and as an additive to improve electrical properties and dimensional stability. Due to its high stability, it can be considered as a replacement for industrially exploited inorganic fillers such as talc and calcium carbonate. Furthermore, the incorporation of biochar into biodegradable plastics can also improve the compost or soil environment. Hussain et al. [34], Bikbulatova et al. [35], Wang et al. [36] and Laird et al. [37] reported increases in both the soil water content and nutrient retention when biochar was used. Biochar prevents soil from acidification (caused, for example, by nitrogen fertilisers) and its porous structure increases microbial activity [38,39,40].

Previous studies have analysed the effect of biochar in PLA on mechanical properties. Bajwa et al. [41] did not observe an increase in the Young’s modulus or tensile strength when biochar was used in PLA. On the contrary, there was a decrease in the relative elongation and impact properties. Kane et al. [42] observed some increase in the strength and modulus of elasticity when biochar was incorporated into PLA. However, there was a significant increase in brittleness and a decrease in thermal stability. Kane et al. [43] declared that the properties of PLA biocomposites depend on the amount of biochar used. When 5 wt.% biochar and lower was applied, PLA biocomposites showed an increase in impact toughness (a critical parameter for many applications) and only a slight decrease in thermal stability. However, the application of 10 wt.% biochar again resulted in significant decreases in the discussed properties. Nevertheless, Zouari et al. [44] reported a decrease in the ultimate strength and modulus of elasticity, as well as the thermal stability, at 5 wt.% biochar concentration in PLA. Hernandez-Charpak et al. [45], Aup-Ngoen et al. [46], and Pudelko et al. [47] showed that the mechanical, structural and thermal properties of the composite are strongly dependent on the raw material used for biochar production. This is fundamentally influenced by the carbon content and the specific surface area of the obtained biochar. Das et al. [48] reported that the higher carbon content and higher specific surface area of biochar increase the strength and modulus of the composite. Another important aspect is the particle size. As the particle size of biochar decreases, the specific surface area generally increases. In contrast, Zhang et al. [49] observed a decrease in the specific surface area with decreasing biochar particle size. This decrease can be attributed to the destruction of the porous structure of bamboo during its grinding.

Although some published results have shown an improvement in mechanical properties, the problem of high brittleness without sacrificing the biodegradability of PLA-based formulations remains. Therefore, the commercial use of these materials is limited to applications that do not require large plastic deformation of articles. Even more limiting is the slow crystallisation rate, which results in products with low heat resistance. The present work addresses all of the above issues and investigates the effect of both the biochar particle size and concentration, along with the impact modifier’s influence on the resulting properties of modified PLA. In addition, the effect of in-mould annealing is analysed to see how the addition of both additives increases the crystallisation rate under different processing conditions and how they improve heat resistance. Another very important aspect that has not been adequately analysed in PLA biocomposites containing biochar is the influence of ageing. It is well known that physical ageing significantly affects the properties and behaviour of PLA during its service life. Therefore, the changes in properties were monitored and analysed not only as a function of the added additives and different processing conditions but also as a function of accelerated ageing’s influence. The novelty of this study is that it provides a comprehensive and previously unpublished review of a variety of factors that can significantly influence the resulting functional properties of PLA biocomposites containing biochar. 

## 2. Materials and Methods

The commercial 100% bio-based PLA Luminy L 130 was purchased from Total Energies Corbion (Gorinchem, Netherlands). Luminy L 130 is a high heat (the melting point is 175 °C, glass transition temperature is 60 °C), medium flow PLA (melt flow index 23 g∙10^−1^ min, ISO 1133-A [50]) homopolymer with minimal 99% L-isomer stereo-chemical purity. Furthermore, the impact modifier MOC 006 (NaturePlast, Mondeville, France) based on PLA with alkylsulfonate, which fulfils the European Regulation on materials and articles intended to come in contact with food (1935/2004/EC [51]; 1169/2004/EUC [52]), was used. The used biochar was produced from agricultural plant waste (corn stover) by high-temperature pyrolysis at 550 °C/120 min under a vacuum atmosphere at a heating rate of 10 °C/min.

### 2.1. Grinding of Biochar

Grinding of the biochar was performed to achieve different particle fragments. The Masuko Sangyo supermasscolloider ultra-fine friction grinder MKCA6-5 (Masuko Sangyo Co., Ltd., Kawaguchi-city, Japan) was used for this purpose. The production process consisted of the uniform dispersion of 500 g of biochar in 10 L of distilled water and continuous grinding while the non-porous grinding stones were in mutual contact. To achieve the low dispersion of particle sizes, the process was repeated 25 times. Consequently, the total grinding time was 20 h. After the milling was completed, the distilled water was removed due to the difference in density of both materials. Further, the rest of the water content was evaporated in a Venticell 22 temperature chamber (MMM Group, München, Germany) at 80 °C for 48 h. Another grinding of the pyrolysed biochar was performed to determine the effect of the biochar particle size on the properties of the PLA biocomposite. The dispersion process of biochar (500 g/10 L) in distilled water was the same as in the previous milling. The difference was in the gap setting of the ceramic stones, where a distance of 150 µm was applied. The grinding process was also repeated 25 times. However, the longer distance of the ceramic stones caused only a 4 h production time. The process of separating distilled water from the ground biochar was analogous to the previous one.

### 2.2. Biochar Properties

As the chemical composition of the biochar (especially the carbon and ash content) is a crucial factor in the future properties of the PLA biocomposite, the chemical composition was first determined using an Agilent ICP-OES 5900 SVDV optical emission spectrometer (Agilent Technologies, Inc., Santa Clara, CA, USA). The nitrogen and carbon contents were determined using a PRIMACS analyser (Skalar, Breda, The Netherlands). The content of mercury (Hg) was determined by thermal decomposition, amalgamation and atomic absorption spectrometry on an AMA 254 analyser (Altec, Prague, Czech Republic). The other elements were determined after extraction in royal meadowsweet using inductively coupled plasma atomic emission spectroscopy (ICP-OES, Thermo Fisher Scientific Inc, Waltham, MA, USA) using same optical emission spectrometer.

Further, the size and distribution of the ground biochar were determined using a laser diffraction analyser LA-920 (Horiba, Kyoto, Japan) and SEM images. As already stated, the specific surface area of the additive is a fundamental property. Consequently, the specific surface area was determined by the Brunauer–Emmett–Teller (BET) theory using Autosorb iQ MP (Quantachrome Instruments, Boynton Beach, FL, USA).

### 2.3. Preparation of PLA Biocomposites

Before production, any moisture was removed from the PLA pellets using the vacuum oven VD53 (Binder, Tuttlingen, Germany) at 50 °C for 12 h. The production of the 1BA (ISO 527 [53]) standard test specimen and the test specimen defined by ISO 179-1 [54] was carried out using a microcompounder MC 15 HT (Xplore Instruments BV, Sittard, The Netherlands) with conical screws at a speed of 100 rpm and a constant temperature profile of 190 °C. The dispersion and distribution were controlled by the level of torque. Neat PLA usually demonstrates a half-time crystallisation (t1/2) in the range of 20–40 min, which is unacceptably long for industrial production, especially for injection moulding, where a typical cycle time is commonly in range from 30 to 90 s [55]. Furthermore, it is known that the crystallisation temperature for PLA is approximately around 100 to 130 °C [7,56]. However, a temperature of the mould of around 40 °C is more often used for industrial production. Hence, a different PLA structure and properties are expected through varying production processes. Therefore, to evaluate the effect of the production process on the properties of PLA biocomposites, samples were injected into the mould at two different heat removal rate settings. The mould temperature (T) of 40 °C and 30 s cooling time were used for intensive heat removal. Contrarily, a mould temperature of 100 °C and 90 s of cooling were used for the excessive cooling variant (in-mould annealing). The additives with a small size and large specific surface area are generally added in a low dosing range (0.5 to 5 wt.%) [57,58,59]. Therefore, the effect of the content of biochar on the PLA biocomposite properties was analysed at 2 and 5 wt.%. The incorporation of 10 wt.% of MOC 006 impact modifier (IM) was also analysed to eliminate the constraining factor of the high brittleness of PLA. The choice of amount is in line with our previous unpublished experiments. The individual compositions of the prepared biocomposites are listed in Table 1.

### 2.4. Accelerated Ageing

The accelerated ageing was performed in a SUN 3600 climatic chamber (Vötsch Industrietechnik, Reiskirchen-Lindenstruth, Germany) using two 4 kW metal halide lamps. The ageing conditions were applied with reference to DIN 75 220. All the samples were conditioned at 25 °C and 50% relative humidity for 240 h before exposure to a constant radiation intensity of 1000 W/m^2^ for 240 h. The temperature of the chamber was set at 28 °C and the relative humidity was set at 65%, which is the average value in the summer months in the Czech Republic [53].

### 2.5. Differential Scanning Calorimetry (DSC)

The thermal properties were analysed using a DSC 1/700 calorimeter (Mettler Toledo, Greofemsee, Switzerland) in a first heating cycle. Samples of approx. 6 mg were heated from 0 to 200 °C with a heating rate of 10 °C∙min^−1^. The DSC chamber was set at a constant nitrogen flow rate of 50 mL∙min^−1^. The glass transition temperature (T_g_), cold crystallisation temperature and enthalpy (T_sc_, ∆H_sc_), pre-melt crystallisation temperature and enthalpy (T_pc_, ΔH_pc_) and melting temperature and enthalpy (T_m_, ∆H_m_) were evaluated. The degree of crystallinity (X_C_) was determined according to the following equation:

(1) $$X_{c}=\frac{{∆H}_{m}-{∆H}_{sc}{-∆H}_{pc}}{{∆H}_{m}^{0}\cdot w_{m}}$$

where (${∆H}_{m}^{0}$) is the melting enthalpy of 100% crystalline PLA (106 J/g) [60] and (w_m_) is the mass fraction of PLA in the composite. The results were evaluated from 3 measurements. Therefore, the standard deviation has not been specified, only the average values are provided.

### 2.6. Mechanical Properties

The tensile properties were measured according to ISO 527 [53] using TiraTest (Labortech, Opava, Czech Republic) with an MFX 500-B extensometer (Mess & Feinwerktechnik GmbH, Velbert, Germany). The tensile modulus (E_t_) was determined at a crosshead speed of 1 mm∙min^−1^, and the tensile strength (σ_m_) and elongation at break (ε_tb_) were measured at a crosshead speed of 5 mm∙min^−1^ as the average of 10 measurements.

The impact properties were measured according to ISO 179/1eU [54] using a Resil 5.5 (Ceast, Torino, Italy) testing machine. For each batch, 10 specimens were broken using a pendulum with a nominal energy of 5 J and an impact velocity of 2.9 m∙s^−1^.

### 2.7. Vicat Softening Temperature (VST)

The heat resistance was determined following the ISO 306 [61] standard using HDT/Vicat 6–300 Allround (Zwick/Roell, Ulm, Germany). Method B120, using a force of 50 N and a heating rate of 120 °C·h^−1^, was used. The measurements of all the specimens were performed before and after the accelerated ageing process to assess the influence of the life cycle phase of the product. The results were evaluated from 3 measurements. Therefore, the standard deviation has not been specified, only average values are provided.

### 2.8. Scanning Electron Microscopy (SEM)

The structure of the fabricated specimens was observed using an FE-SEM TESCAN MIRA 3 (Tescan, Brno, Czech Republic) instrument with an accelerated voltage of 10 kV. Prior to analysis, the samples were coated with 6 nm of platinum/palladium (Pt/Pd) alloy (80/20) using a Leica EM ACE200 sputter coater (Leica Microsystems, Wetzlar, Germany).

## 3. Results

### 3.1. Chemical Compositions of Biochar

The results of the chemical composition of the pyrolysed biochar are listed in Table 2. A 46.7% carbon content characterises biochar. This value corresponds to the raw material used. When using woody raw materials (high cellulose content), higher carbon values (from 50 to 70%) are generally achieved [62,63,64]. On the contrary, when waste raw materials such as sludge are used, the carbon content is commonly lower [65]. However, the carbon content of most biochar is commonly just in the range from 40 to 50% [66,67,68,69,70]. The high ash content of biochar corresponds to the applied pyrolysis conditions. High-temperature pyrolysis generates higher amounts of ash than low-temperature pyrolysis (<500 °C) [64,71]. The content of heavy metals does not exceed the limit criteria for materials intended for composting (CSN 465735 [72], Decree 273/2021 Coll. [73]).

### 3.2. Ground Biochar Properties

**Figure 1:**
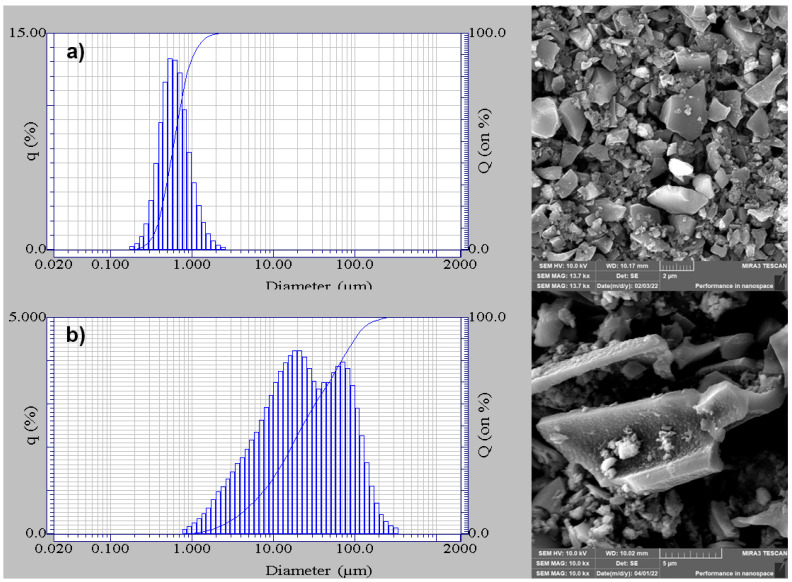
Biochar particle size distribution (**left**) and SEM images (**right**)—(**a**) B1 and (**b**) B2.

The determined particle size and distribution of the ground biochar are shown in Figure 1a,b. Biochar produced at a zero gap in the ceramic stones was characterised by an average particle size of 0.6 µm and a relatively narrow distribution of particles with sharp, regular shapes. Increasing the gap to 150 µm resulted in greater particle size anisotropy. The ground biochar contained long lamellar fragments and small regular particles. The evaluated average particle size was 41.1 µm. The results of the specific surface area of the ground biochar confirm the theoretical assumptions about the effect of the biochar particle size. A specific surface area of 52 m^2^·g^−1^ was evaluated for biochar with an average particle size of 41.1 µm and a specific surface area of 118 m^2^·g^−1^ for biochar with an average particle size of 0.6 µm.

### 3.3. Differential Scanning Calorimetry (DSC)

**Figure 2:**
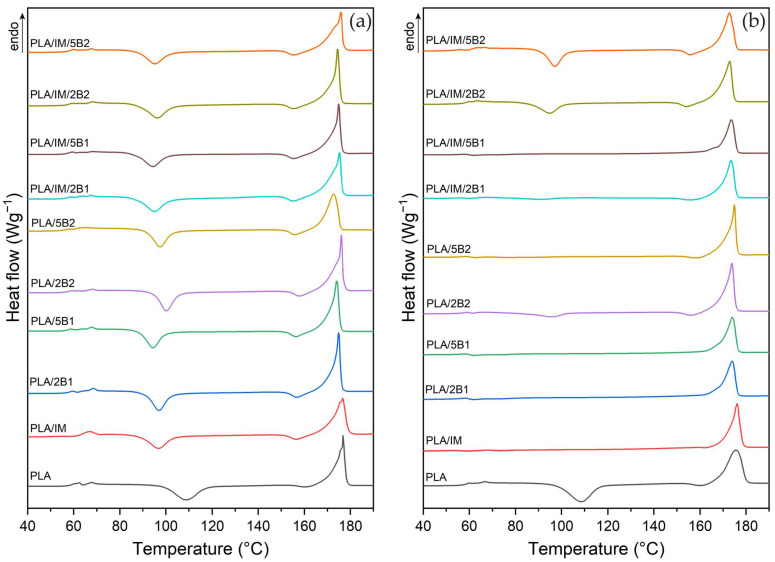
First heating DSC curves of PLA samples injected into the moulds with the following annealing conditions: (**a**) T = 40 °C/30 s, and (**b**) T = 100 °C/90 s.

The results of the first non-isothermal heating of the as-produced PLA-based samples are shown in Figure 2 and listed in Table 3. The glass transient temperatures were used for neglecting the technological processing aspects evaluated from the second heating cycle. An increase in the melting enthalpy (∆Hm) and degree of crystallinity (Xc) is evident from the evaluated results of the as-produced neat PLA specimens after increasing the mould temperature (T) and cooling time from 40 °C/30 s to 100 °C/90 s. Also, Aliotta et al. [74] presented the effect of the mould temperature and cooling time on the morphology of PLA. When the mould temperature was set at 110 °C and the cooling time was increased from 30 to 60 s, an increase in the degree of crystallinity from 19.2 to 21.5% was observed. Harris et al. [55] reported a significant increase in the degree of crystallinity (from 10 to 43%) when producing PLA at a mould temperature of 110 °C compared to 25 °C. However, the presented increase was multiple times higher than for PLA Luminy L130 when similar mould temperatures were used. This could be caused by PLAs different crystallisation abilities.

The incorporation of the impact modifier (IM) evoked a shift in cold crystallisation toward lower temperatures and a slight increase in the degree of crystallinity of the PLA injected into the mould at 40 °C when compared to neat PLA. Furthermore, in-mould annealing of the PLA/IM samples at 100 °C and 90 s favoured melt crystallisation and, as a result, the disappearance of the cold crystallisation peak could be observed. Due to such conditions, an increase in the degree of crystallinity from 13.3% to 42.5% has been found for in-mould annealed PLA/IM samples compared to cold mould processing. The addition of 2 wt.% biochar with an average particle size of 0.6 µm (B1) to PLA under “cold mould” conditions (40 °C, 30 s) caused the same shift in the Tcc to lower temperatures as IM, demonstrating that both are capable of increasing the nucleation density. However, only minor changes in crystallinity were observed when compared to pure PLA. On the other hand, high-temperature annealing and a prolonged cooling cycle (100 °C/90 s) resulted in a high degree of crystallinity (39%) of the PLA/2B1 composite. Increasing the biochar (B1) content to 5 wt.% caused a slight shift in the Tcc and provided more nucleation sites for crystallisation. The degree of crystallinity increased from 8.8% (2 wt.% B1) to 12.0% (5 wt.% B1) at 40 °C mould temperature and 30 s cooling time and from 39.0% (2 wt.% B1) to 42.3% (5 wt.% B1) when the 100 °C and 90 s cooling was used, see Table 3. The biochar with an average particle size of 41.1 µm (B2), at a concentration of 2 wt.%, caused a minor increase in the degree of crystallinity compared to the incorporation of smaller-sized biochar when the more intensive heat removal (40 °C/30 s) was applied. Contrarily, the higher mould temperature and longer cooling time evoked a notably lower structural order increase (X_c_ = 30%) than biochar with a smaller particle size (39%). When the B2 content was increased to 5 wt.%, a reduction in the cold crystallisation enthalpy (∆Hcc) and the pre-melting enthalpy (∆Hpc) was observed when “cold mould” processing was carried out. Therefore, a degree of crystallinity up to 40% was also observed for this material variant at a mould temperature of 100 °C and a cooling time of 90 s. Consequently, it could be stated that the influence of the production process conditions is a crucial issue for the structural order of the PLA biocomposite. A similar conclusion was estimated in several other studies [7,75,76,77], where the variation of the nucleating additives in concentration ranges from 1 wt.% to 5 wt.% caused only a slight increase in the degree of crystallinity of PLA at a mould temperature around 25 °C, while a temperature of around 100 °C caused an enormous increase in the structure order. Another aspect that has to be considered is the structure form. Depending on the crystallisation conditions of PLA, ordered α-phases and disordered α′- metastable structures can be simultaneously formed [78]. These structural inhomogeneities subsequently affect the energy required to melt the crystals (different polymorphs) and result in double peak behaviour [7]. In our investigation, only neat PLA and PLA with an impact modifier showed a lower temperature shoulder in the intensive cooling process (40 °C/30 s). This phenomenon is generally attributed to the melting, recrystallisation and re-melting mechanism of semicrystalline polymers that exhibit polymorphism [79]. The phenomenon disappeared when biochar was incorporated and when the mould temperature and cooling time were increased as well as when biochar was incorporated.

The glass transition temperature did not change when the different biochar sizes, contents, and production processes were applied. However, the addition of biochar evoked a decrease in the melt temperature and cold crystallisation temperature. The temperature shift increases with the biochar content and higher mould temperature. However, no differences in the influence of the particle size were found. Also, Zhang et al. [49] presented similar dependence for PLA with biochar pyrolysed from bamboo with a characteristic particle size range from 163 to 28.7 µm. Any size of this used biochar characteristic with a surface area of 63 to 561 m^2^∙g^−1^ did not ensure the nucleation effect changes caused by the short injection moulding production process. The intensive cold removal could also be ascribed to the poor nucleation effect of biochar from digested dairy manure (surface area 19.3 m^2^∙g^−1^) and white pine wood chip (surface area 47.4 m^2^∙g^−1^) with an average particle size of 25 µm presented by Hernandez-Charpak et al. [45]. Contrarily, Arrigo et al. [59] significantly increased the crystallinity degree of PLA with the addition of 1 wt.% biochar from coffee powder with a particle size of 10 µm. However, a different production process (lab press cooling) with a 170 °C/120 s production condition was applied, unfortunately without information about the cooling. Similar to our results, a further increase in the biochar content to 5 wt.% did not evoke a further structure order increase.

The addition of 10 wt.% of impact modifier to PLA with biochar (2 and 5 wt.%) induced significant changes in the results discussed. The considerable increase in the structural order (compared to PLA with biochar) was evaluated only for biochar with an average particle size of 0.6 µm, ensured by a 40 °C mould temperature and 30 s cooling process adjustment. The ternary biocomposites with a higher amount of biochar and biochar with an average particle size of 41.1 µm showed no nucleation improvement. Contrarily, a significant decrease in the crystallinity degree was evaluated when the impact modifier was used for this material variant under slower heat removal process conditions (100 °C/90 s). Melt crystallisation was hindered by the combination of IM and B2 at both levels. This phenomenon could be attributed to the size of B2, which, in combination with IM, constrained the PLA macromolecular chains and resulted in lower content formation of the crystalline phase [59]. 

**Figure 3:**
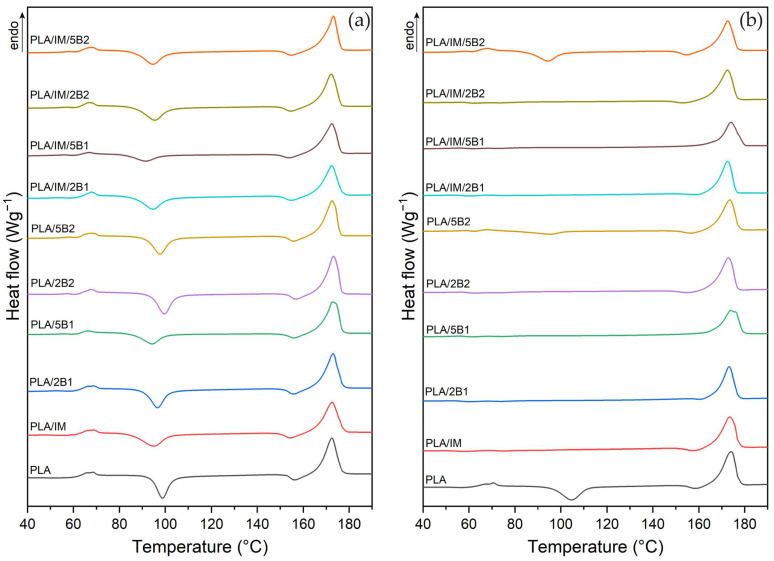
First heating DSC curves of aged PLA samples injected into the moulds with the following annealing conditions: (**a**) T = 40 °C/30 s, and (**b**) T = 100 °C/90 s.

**Figure 4:**
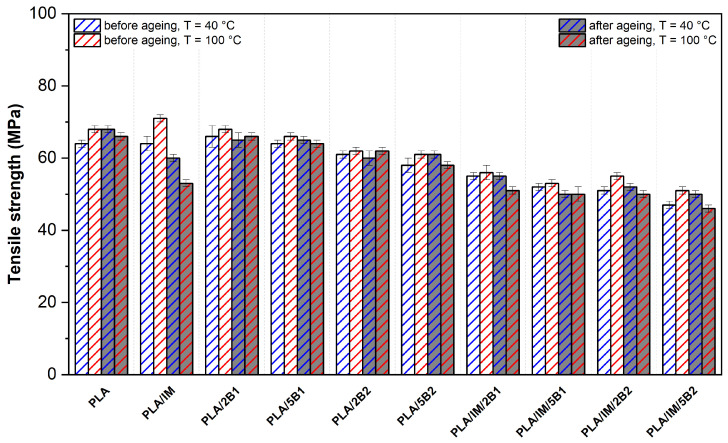
Tensile strength of as-produced and aged PLA samples injected into the moulds with different annealing conditions.

The results of the first non-isothermal heating of the as-produced PLA samples are shown in Figure 3 and listed in Table 4. PLA has a relatively high resistance to photodegradation (UV radiation) [80,81]. On the other hand, it is well known that PLA is extremely sensitive to the hydrolysis phenomenon, which starts to accelerates in the glass transition temperature region. Hydrolysis of PLA causes the scission of ester bonds, which evokes decreases in the molecular mass of the material, physical and structure changes as well as changes in the mechanical properties. Hence, the issue of ageing is most often studied only under different temperatures and humidity conditions. Cui et al. [82] and Müller et al. [83] evaluated the decrease in the glass transition temperature of standard test specimens (ISO 527-1A [53]) of neat PLA when physical ageing at 23 °C and 50% relative humidity for 720 h was applied. On the contrary, Lesaffre et al. [84] did not observe any significant changes in the glass transition temperatures and melting temperatures after 60 days of exposition to 50 °C and 75% relative humidity. Also, Rocca-Smith et al. [85] presented similar conclusions within the ageing of neat PLA under 50% relative humidity and a temperature of 50 °C in a microclimate chamber, where no changes in the transient temperatures, structure order, and molecular weight were evaluated. However, a further increase in the moisture content evoked a significant decrease in the molecular weight and transition temperatures, and it increased the crystallinity degree. The ageing process of neat PLA evoked only a slight decrease in the cold crystallisation temperature (T_cc_), pre-melt crystallisation temperature (T_pc_) and melting temperature (T_m_) in both process adjustment (Figure 4 and Table 4). However, the glass transient temperature (T_g_) and crystallinity degree (X_c_) were unchanged despite the fact that some changes in enthalpies have been recorded. A similar dependence was established for PLA with an impact modifier.

Due to its hydrophilic properties [34,35,36], biochar can induce an increase in hydrolysis degradation during the ageing process. On the contrary, it can also cause a UV stabilising effect. The aged PLA samples with biochar did not evoke any shifts in the T_g_. Therefore, neither hydrolytic nor photodegradation can be assumed. This is probably due to the choice of a long irradiation ageing programme at a lower temperature and humidity (28 °C, 65%). The production of PLA with 2 wt.% and 5 wt.% of biochar, with an average particle size of 0.6 µm, at a higher mould temperature and longer cooling time (100 °C/90 s) enables the achievement of a highly structured system. This is probably why no changes in the degree of crystallinity occurred during the ageing process. However, the ageing process samples produced under more intensive heat removal (40 °C/30 s) caused an increase in the crystallinity degree (physical ageing). The PLA biocomposites with biochar of 41.1 µm average particle size showed different dependence. The considerable changes in the aged PLA/2B2 specimens were evaluated within samples produced at a 100 °C mould temperature and 90 s cooling. Nevertheless, a decrease in the crystallinity degree was evaluated for the aged PLA/5B2 samples produced with the same process adjustment. Similar to other material variants, accelerated ageing did not affect the transition temperatures of the PLA ternary composites. The crystallinity degree of the aged PLA/IM/B1 samples produced at a higher mould temperature and longer process time adjustment increased from 36.3% to 41.2% and 45.5%, respectively, if a higher amount of biochar was used. If the higher heat transfer was applied for PLA/IM/5B1, an even higher increase in the structure order was evaluated due to the ageing process. The ternary composite containing biochar of 41.1 µm average particle size did not show major changes in the crystallinity degree. The only exception was PLA/IM/2B2, which was produced at a high mould temperature and cooling time adjustment. An enormous increase from 18.2% to 43.4% was observed after accelerated ageing. 

### 3.4. Mechanical Properties

**Figure 5:**
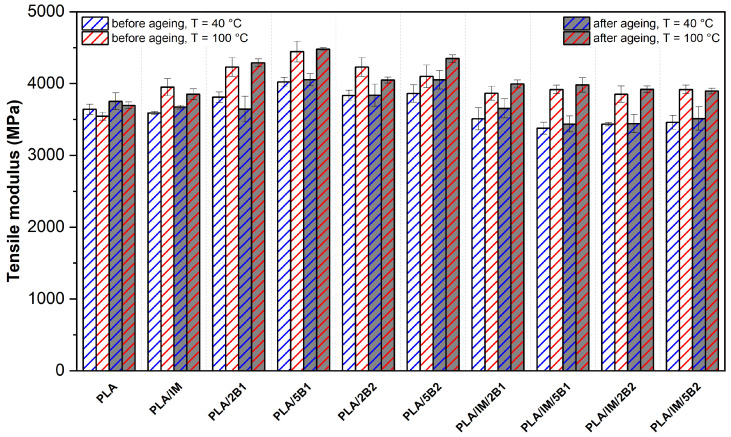
Tensile modulus of as-produced and aged PLA samples injected into the moulds with different annealing conditions.

**Figure 6:**
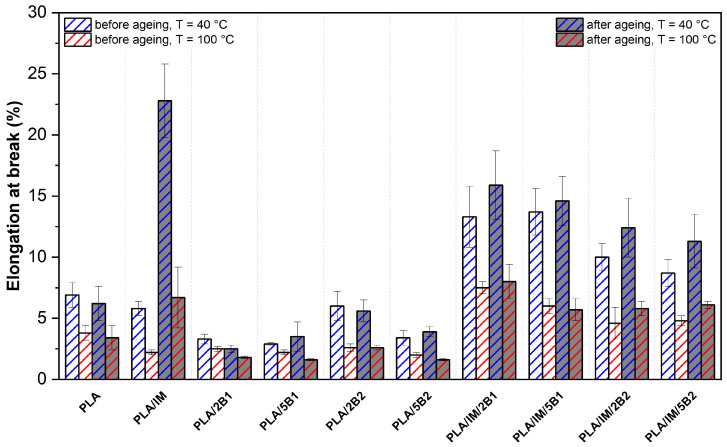
Elongation at break of as-produced and aged PLA samples injected into the moulds with different annealing conditions.

The results of the mechanical properties of the as-produced and aged PLA samples are shown in Figure 4, Figure 5 and Figure 6. The considerable influence of the production process adjustment on the structure of neat PLA was observed in the DSC analysis. The higher mould temperature and longer process time (100 °C/90 s) allowed an increase in the crystalline degree. The crystalline structure had enough time for growth, which caused a decrease in the tensile strength and elongation at break compared to the more intensive heat-removing process variant (40 °C/30 s). Similarly, Vadori et al. [86] observed a decrease in elongation when a 90 °C mould temperature was used during injection moulding. Nevertheless, a meaningful tensile strength increase was evaluated. The reason for these differences could be the different production times as well as different crystallisation kinetics of the used PLA system (Ingeo 3801X, NatureWorks, Plymouth, MI, USA). Schäfer et al. [75] confirmed the assumption about the influence of the production time on the structure formation. Both mechanical properties (tensile strength and elongation at break) significantly decreased if the mould temperature and process time of injection moulding production increased from 25 °C/55 s to 105 °C/455 s. However, like the previous author, the influence of the process parameters on the mechanical properties was analysed on a modified PLA system.

Incorporating an impact modifier into PLA evoked an increase in the tensile strength and a decrease in the modulus. The same results have been observed by Scaffaro at al. [87] using the impact modifier IMS550 by Sukano at different contents (4–6 wt.%). Furthermore, in-mould annealing of PLA/IM caused a significant increase in the degree of crystallinity (see Table 3). This evoked a significant rise in the tensile modulus and decreased the elongation at break.

PLA/2B1 showed meaningful changes in the tensile strength and modulus only when a higher mould temperature and longer production times were used (100 °C/90 s). Compared to the 40 °C/30 s production variant, the tensile modulus increased from 3811 MPa to 4230 MPa, the tensile strength remained unchanged and the elongation at break dropped from 3.3% to 2.5%. Using 5 wt.% of B1 biochar (average particle size of 0.6 µm) evoked a considerable increase in the tensile modulus, even with the more intensive heat production process adjustment, while a decrease in the tensile strength and elongation was observed compared to a lower amount of biochar in the PLA. Arrigo et al. [59] observed a significant increase in the tensile modulus and minimal changes in the tensile strength even at a low concertation (2.5 wt.%) of biochar with an average particle size of 10 µm in the PLA matrix. The reason for this phenomenon may be, as in our results, a technological aspect (influence of the temperature and heat transfer on the structure arrangement). The PLA biocomposite samples were produced by compression moulding (170 °C/120 s) and solvent casting (20 °C/24 h). Unfortunately, information about the cooling conditions was not provided. The incorporation 2 wt.% of biochar of an average particle size of 41.1 µm (B2) to PLA did not evoke any significant changes in the tensile modulus and elongation at 40 °C/30 s process setting. On the other hand, the higher mould temperature and longer production time showed meaningful changes. With respect to the standard deviation of the evaluated results, the increased B2 content (5 wt.%) caused only a decrease in elongation. However, Zouari et al. [44] reported a significant increase in the tensile modulus and minimal changes in the tensile strength of PLA biocomposite containing 5 wt.% of biochar made from beechwood with an average particle size of 75 µm and surface area 230 m^2^·g^−1^. Biochar made from beechwood was characterised by a higher specific surface area, causing, due to the improved interface between the matrix and the BC, greater changes in the mechanical properties of PLA. This result shows the importance of the raw material used for the production of biochar. Biochar made from woody raw materials is characterised by a higher carbon content, a higher specific surface area and a lower ash content (beech wood biochar had carbon content 77.8% and 1.8% ash content).

The evaluated results of the tensile strength show more clearly the differences in the effect of the biochar size (B1, B2) at both concentrations. Hence, biochar with an average particle size of 0.6 µm (PLA/B1) evoked a minimal drop in the PLA strength, while a significant decrease was observed for PLA biocomposite with biochar of 41.1 µm average particle size (PLA/B2). The reason for these differences is the different interaction of the biochar with the PLA matrix. The biochar B1 was characterised by a significantly higher specific surface area than B2 biochar with higher particle size. Therefore, it is plausible that the incorporation caused stronger particle–matrix interface adhesion. In addition, the results suggest that the submicron size of the particles may be below the critical defect size, which, together with their good dispersion and distribution in the PLA matrix, may be responsible for this phenomenon. Also, Mozrall et al. [88] evaluated the impact of the biochar particle size on the mechanical properties. The incorporation of biochar of a 10 µm average particle size simultaneously evoked at a 5 wt.% concentration in PLA increases in the tensile strength and modulus. On the contrary, incorporating biochar of a higher particle size (250 µm and 400 µm) evoked an enormous decrease in the tensile strength. A similar dependence was presented by Zhang et al. [49], where the tensile strength and modulus continuously decreased with an increasing biochar size (60–500 µm) of PLA biocomposite.

All the impact-modified PLA–biochar composites showed decreased tensile strength in both production variants when compared to virgin PLA. The decrease in strength increased with the amount of biochar. A similar dependence was estimated for the tensile modulus. It must be said here that the decline is only marginal, especially at low mould temperatures and short production times. The incorporation of both additives simultaneously also caused a significant increase in ductility.

From the above results, it is clear that both the impact modifier alone and the biochar increase the degree of crystallinity of PLA formulations (see Table 3). However, the simultaneous use of both additives in the PLA biocomposite does not cause a significant change in the degree of crystallinity according to the results of the DSC analysis. Nevertheless, there may be differences in the shape and size of the ordered structures. Due to the higher number of heterogeneous nucleation sites, the structure could be characterised by a high number of small spherulites, which, together with the functional component of the impact modifier, could induce an increase in elongation. According to the tensile strength results, the structure formed is not able to transmit such strength loads as in the PLA biocomposites containing a separate component and premature failure is observed. The influence of the process adjustment and biochar particle size is also evident from the observed results of the impact-modified PLA–biochar composites. Similar to previous results, a higher mould temperature and longer production time increased the degree of crystallinity of the PLA ternary biocomposites. The PLA/IM/B1 biocomposites at both concentrations showed a lower decrease in tensile strength and a higher increase in elongation. The results are particularly evident at a higher biochar amount and lower heat dissipation production variant (40 °C/30 s). These phenomena could also be attributed to the influence of the biochar particle size on spherulite growth. A smaller biochar size produces a higher ratio of heterogeneous nucleation sites compared to the same weight content of biochar characteristic of a larger particle size, which further allows the formation of a fine structure. The result is the higher resistance of these materials to fracture propagation.

The accelerated ageing process did not induce any meaningful changes in the mechanical properties of neat PLA. With respect to the standard deviations of the evaluated results, no changes were recorded for the PLA–biochar composites during both production settings. Nevertheless, significant changes in the properties of the impact-modified PLA were observed. The tensile strength of PLA/IM produced under 40 °C/30 s process conditions decreased from 71 MPa to 60 MPa and from 64 MPa to 53 MPa for the PLA/IM sample at the higher mould temperature. Furthermore, a significant increase in ductility and slight increase in the tensile modulus were observed when PLA/IM was exposed to ageing conditions. On the contrary, incorporating biochar into PLA with an impact modifier did not evoke any significant changes in the mechanical properties.

**Figure 7:**
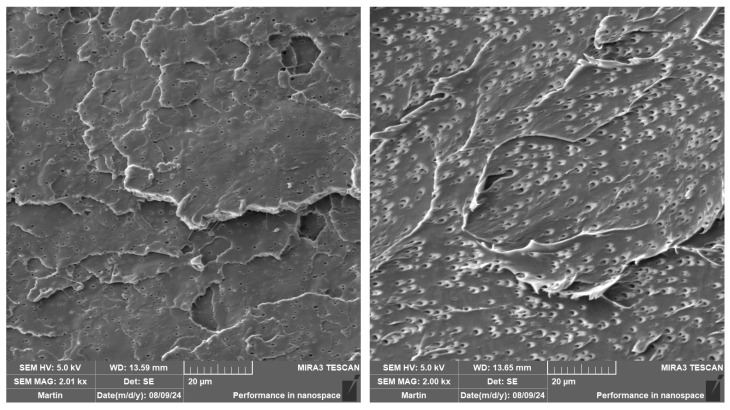
SEM images of as-produced PLA/IM samples injected into the moulds with the following annealing conditions: (**left**) T = 40 °C/30 s, and (**right**) T = 100 °C/90 s.

**Figure 8:**
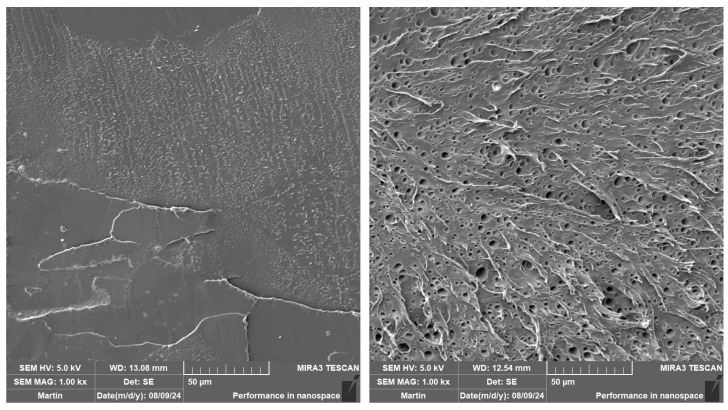
SEM images of aged PLA (**left**) and PLA/IM (**right**) injected into the moulds with T = 100 °C and 90 s annealing.

**Figure 9:**
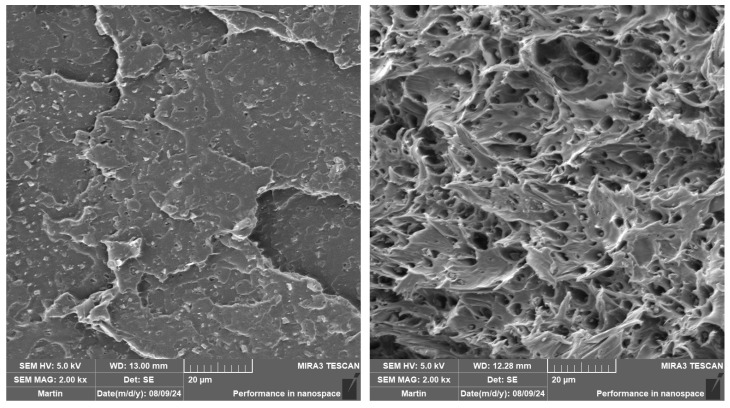
SEM images of as-produced PLA/5B1 (**left**) and PLA/IM/5B1 (**right**) injected into the moulds with T = 40 °C and 30 s annealing.

The impact strength of the as-produced and aged PLA samples is listed in Table 5. The expected increase was observed when the impact modifier was applied, especially at a high mould temperature (see Figure 7). This can be attributed to the observed nucleation effect of IM, where smaller spherulites were formed at more dense nucleation sites (see Table 3 and Table 4). Furthermore, Scaffaro et al. [87] observed the same increase using the Sukano IMS550 (Sukano, Schindellegi, Switzerland) impact modifier (8 wt.% loading). They observed the formation of micro-holes where the impact modifier accumulates during phase separation. Exactly the same behaviour can be seen in our SEM investigations in Figure 8. Incorporation of biochar into the PLA matrix did not result in any significant changes in the impact strength at more intensive heat dissipation production (40 °C/30 s). The only exception was PLA/5B1, where a slight increase in toughness was observed. This phenomenon could be linked to the size (below critical defect size of 1 µm) and uniform distribution of B1 in the PLA matrix (see Figure 9). On the contrary, applying the higher mould temperature and longer production time ensured an increase in the impact strength of all the composites. The results show the best toughening effect is obtained at low additions and low particle sizes of biochar (PLA/2B1), from 17 to 28 kJ∙m^−2^. The same conclusions were reached by Kane et al. [43], who investigated the impact strength of PLA composites at different biochar contents processed at a mould temperature of 90 °C. The highest increase was achieved at a biochar content of 2.5 wt.%, while the average particle size was 10 µm.

The addition of biochar together with an impact modifier to the PLA matrix affected the impact properties as a function of the biochar size. The bigger particles (B2) behave as a higher stress concentrator, which is a well-known phenomenon. At a low mould temperature, there is no significant impact of the biochar particles on the toughness. Nevertheless, a synergistic effect was observed for PLA/IM/5B1, where the impact strength increased up to 95 kJ/m^2^. The fractured surfaces of PLA/5B1 and PLA/IM/5B1 can be observed in Figure 9. In contrast, the biochar incorporation increased the impact strength at a high mould temperature.

The results of the accelerated ageing did not show any significant changes in the impact strength of the PLA biocomposite with biochar (except PLA/5B2). However, for the PLA ternary composites, there is a trend toward increased toughness, especially at a high mould temperature, which is consistent with the results of the DSC analysis.

### 3.5. Vicat Softening Temperature (VST)

The VST of the PLA composites with an impact modifier and biochar before and after artificial ageing are shown in Table 6. The addition of biochar, as well as the incorporation of an impact modifier, did not affect the VST under more intensive heat removal production (40 °C/30 s). The VST of the neat PLA was not affected even when processed by injection moulding using a higher mould temperature (100 °C).

Nevertheless, the heterogenous nucleation caused by the addition of biochar or an impact modifier combined with a higher mould temperature and cooling time promoted the crystallisation of PLA; therefore, the VST of the composites exhibits a considerable increase. The incorporation of biochar B1 to the PLA evoked an enormous increase in the impact strength even at a lower concentration (2 wt.%). From the VST results of the ternary composites, the influence of the particle size is also obvious. Impact-modified PLA-biochar composites with a 41.1 µm average particle size (B2) of biochar show no changes in the VST at both concentrations, while PLA/IM/B1 composites with a 0.6 µm average particle size reached a VST of 85.2 °C and 94.6 °C, respectively. This is probably due to the higher specific surface area of B1 and enhanced B1 particle–PLA matrix interface. Similar conclusions were stated by Quian et al. [89] and Xia et al. [90]. The accelerated ageing did not evoke any significant changes in the VST, except in the composites that contain 2 wt.% of B2.

## 4. Conclusions

The evaluated results showed the crucial influence of the production process conditions on the structure order of PLA, PLA–biochar composites, and impact-modified PLA–biochar composites. Due to the intensive heat removal and shorter production time, PLA forms a lower structure order and inhomogeneity. The incorporation of additives and their interaction with the PLA matrix significantly change the crystallinity. However, the impact of production is more fundamental. From the analysis of accelerated ageing, it can be concluded that more suitable thermodynamic conditions for the structure order formation of PLA biocomposites subsequently ensure its long-term stability and resistance to property changes due to physical ageing. This assumption was confirmed, especially by the results of PLA/IM, PLA/2B1, PLA/5B1, and PLA/IM/5B1. On the contrary, the significant physical ageing changes were evaluated for the PLA biocomposites and ternary composites with biochar of a larger particle size.

The influence of the biochar particle sizes was also reflected in the mechanical properties. The differences were obvious in the results of the tensile strength. Biochar with an average particle size of 0.6 µm evoked a minimal drop in PLA strength. On the other hand, a significant decrease was observed for the PLA/B2 biocomposites with an average biochar particle size of 41.1 µm. The increasing content of both biochars (B1, B2) from 2 wt.% to 5 wt.% caused a rise in the tensile modulus and a drop in the elongation. Production at a higher mould temperature and a longer production time further increased the differences between the neat PLA and the PLA–biochar composites. The incorporation of biochar together with an impact modifier into PLA caused an enormous rise in elongation. The high mould temperature and longer cooling time also enhanced the Vicat softening temperature (VST) of the PLA–biochar composites and impact-modified PLA–biochar composites. The biochar characterised by a smaller particle size showed higher increases in the VST.

The accelerated ageing process did not evoke any significant changes in the mechanical properties, impact strength and VST of the neat PLA and only small changes in the bio-composite with the biochar during both production adjustments. Nevertheless, there were considerable changes in the mechanical properties and impact strength of the PLA biocomposites containing an impact modifier. Accelerated aging induced an increase in the toughness of the ternary PLA composites, which was particularly evident at high mould temperatures and longer fabrication times.

## Figures and Tables

**Table 1:** Sample compositions.

Sample Designation	Composition (wt. %)			
	PLA	MOC006	Biochar (B1)— avg. 0.6 µm	Biochar (B2)— avg. 41.1 µm
PLA	100	-	-	-
PLA/IM	90	10	-	-
PLA/2B1	98	-	2	-
PLA/IM/2B1	88	10	2	-
PLA/5B1	95	-	5	-
PLA/IM/5B1	85	10	5	-
PLA/2B2	98	-	-	2
PLA/IM/2B2	88	10	-	2
PLA/5B2	95	-	-	5
PLA/IM/5B2	85	10	-	5

**Table 2:** Chemical composition of the used biochar.

Element	Content (%)	Heavy Metal	Content (mg/kg)
Ash	36.6	Hg	0.006
C	46.7	As	<1.0
O	28.2	Be	<0.1
N	0.8	Cd	<0.05
P	0.7	Co	1.47
K	1.0	Cr	6.7
Mg	0.4	Cu	6.8
Ca	6.7	Mn	136.0
S	0.07	Mo	0.47
		Ni	8.8
		Pb	<5.0
		V	<5.0
		Zn	60.5

**Table 3:** The results of the DSC analysis before accelerated ageing.

Sample	T (°C)	T_g_ (°C)	T_sc_ (°C)	T_pc_ (°C)	T_m_ (°C)	ΔH_sc_ (J/g)	ΔH_pc_ (J/g)	ΔH_m_ (J/g)	X_c_ (%)
PLA	40	59.6	108.7	160.1	176.7	29.30	4.27	39.50	5.6
	100	59.8	108.4	159.8	175.7	27.49	4.07	41.72	9.6
PLA/IM	40	59.8	96.1	156.0	177.4	26.58	5.93	45.19	13.3
	100	60.1	-		175.1	-	-	40.50	42.5
PLA/2B1	40	-	97.1	156.7	174.6	29.64	6.32	45.15	8.8
	100	-	-		173.8	-	-	40.47	39.0
PLA/5B1	40	-	94.6	156.2	173.8	26.48	6.04	44.60	12.0
	100	-	-		173.7	-	-	42.63	42.3
PLA/2B2	40	60.1	100.3	157.8	175.8	27.63	5.62	44.05	10.4
	100	60.3	95.7	156.0	173.5	11.12	4.33	46.62	30.0
PLA/5B2	40	60.0	97.6	155.8	172.5	27.59	5.58	42.35	9.1
	100	60.9	-	158.1	174.7	-	2.58	43.13	40.3
PLA/IM/2B1	40	60.4	95.2	155.1	175.0	25.91	5.77	43.55	12.7
	100	59.9	91.5	155.6	173.3	3.32	2.86	40.03	36.3
PLA/IM/5B1	40	-	94.4	155.3	174.7	25.20	5.80	41.69	11.9
	100	-	-	-	173.5	-	-	38.11	42.3
PLA/IM/2B2	40	59.6	96.2	155.3	174.2	27.91	5.73	42.87	9.9
	100	59.9	94.9	154.2	172.6	20.88	5.06	42.92	18.2
PLA/IM/5B2	40	60.7	95.4	155.5	175.8	25.53	5.34	41.84	12.2
	100	60.9	93.4	154.0	172.6	17.83	4.41	38.25	17.8

T—temperature of mould; T_g_—glass transition temperature; T_sc—_cold crystallisation temperature ∆H_cc—_cold crystallisation enthalpy; T_pc_—pre-melt crystallisation temperature; ΔH_pc_—pre-melt crystallisation enthalpy; T_m_—melting temperature; ∆H_m_—melting enthalpy; X_C_—degree of crystallinity; PLA—neat poly(lactic acid); PLA/IM—10 wt.% of impact modifier, PLA/2B1—2 wt.% of biochar B1; PLA/5B1—5 wt.% of biochar B1; PLA/2B2—2 wt.% of biochar B2; PLA/5B2—5 wt.% of biochar B2, PLA/IM/2B1—10 wt.% of impact modifier and 2 wt.% of biochar B1; PLA/IM/5B1—10 wt.% of impact modifier and 5 wt.% of biochar B1; PLA/IM/2B2—10 wt.% of impact modifier and 2 wt.% of biochar B2; PLA/IM/5B2—10 wt.% of impact modifier and 5 wt.% of biochar B2.

**Table 4:** The results of the DSC analysis after accelerated ageing.

Sample	T (°C)	T_g_ (°C)	T_sc_ (°C)	T_pc_ (°C)	T_m_ (°C)	ΔH_sc_ (J/g)	ΔH_pc_ (J/g)	ΔH_m_ (J/g)	X_c_ (%)
PLA	40	59.8	98.9	156.3	172.0	29.09	5.99	45.88	10.2
	100	59.7	104.6	158.5	174.1	25.77	3.52	44.39	14.2
PLA/IM	40	59.7	95.0	154.5	172.3	28.70	6.43	44.22	9.5
	100	59.6	-	157.6	173.1	-	3.17	44.27	43.1
PLA/2B1	40	-	96.7	155.8	172.6	27.91	6.38	45.60	10.9
	100	-	-	160.1	173.1	-	0.29	40.12	38.3
PLA/5B1	40	-	94.4	155.8	172.4	19.86	5.28	44.68	19.4
	100	-	-	-	173.8	-	-	41.37	41.1
PLA/2B2	40	59.6	99.7	156.7	173.1	29.28	5.89	46.13	10.6
	100	59.9	-	154.8	172.5	-	3.05	45.28	40.7
PLA/5B2	40	58.9	97.9	155.8	172.4	26.21	5.82	43.84	11.7
	100	60.2	94.8	156.3	173.4	11.95	3.65	42.97	27.2
PLA/IM/2B1	40	60.6	94.7	154.7	172.0	26.38	5.48	41.49	10.3
	100	59.9	-	159.0	172.4	-	1.38	39.82	41.2
PLA/IM/5B1	40	-	91.3	154.0	172.1	13.39	4.50	43.41	28.3
	100	-	-	-	173.7	-	-	41.03	45.5
PLA/IM/2B2	40	59.5	95.4	154.5	171.9	27.22	5.41	42.92	11.0
	100	59.6	-	153.0	172.2	-	3.01	43.53	43.4
PLA/IM/5B2	40	60.1	94.7	154.8	173.0	25.24	5.44	41.93	12.5
	100	60.1	94.4	154.6	172.4	19.42	4.99	40.76	18.1

T—temperature of mould; T_g_—glass transition temperature; T_sc—_cold crystallisation temperature ∆H_cc—_cold crystallisation enthalpy; T_pc_—pre-melt crystallisation temperature; ΔH_pc_—pre-melt crystallisation enthalpy; T_m_—melting temperature; ∆H_m_—melting enthalpy; X_C_—degree of crystallinity; PLA—neat poly(lactic acid); PLA/IM—10 wt.% of impact modifier, PLA/2B1—2 wt.% of biochar B1; PLA/5B1—5 wt.% of biochar B1; PLA/2B2—2 wt.% of biochar B2; PLA/5B2—5 wt.% of biochar B2, PLA/IM/2B1—10 wt.% of impact modifier and 2 wt.% of biochar B1; PLA/IM/5B1—10 wt.% of impact modifier and 5 wt.% of biochar B1; PLA/IM/2B2—10 wt.% of impact modifier and 2 wt.% of biochar B2; PLA/IM/5B2—10 wt.% of impact modifier and 5 wt.% of biochar B2.

**Table 5:** Charpy impact strength of as-produced and aged PLA samples injected into the moulds with different annealing conditions.

Sample	As-Produced						Aged					
	40 °C			100 °C			40 °C			100 °C		
PLA	18	±	1	13	±	4	21	±	3	21	±	3
PLA/IM	29	±	2	79	±	2	39	±	7	65	±	12
PLA/2B1	17	±	3	28	±	6	20	±	4	27	±	9
PLA/5B1	21	±	4	21	±	4	23	±	3	18	±	3
PLA/2B2	18	±	2	19	±	3	21	±	2	25	±	4
PLA/5B2	18	±	2	14	±	1	19	±	2	34	±	4
PLA/IM/2B1	37	±	6	49	±	6	53	±	11	52	±	8
PLA/IM/5B1	95	±	8	59	±	8	95	±	13	51	±	9
PLA/IM/2B2	28	±	6	22	±	5	29	±	4	44	±	4
PLA/IM/5B2	29	±	4	19	±	5	18	±	2	34	±	3

PLA—neat poly(lactic acid); PLA/IM—10 wt.% of impact modifier, PLA/2B1—2 wt.% of biochar B1; PLA/5B1—5 wt.% of biochar B1; PLA/2B2—2 wt.% of biochar B2; PLA/5B2—5 wt.% of biochar B2, PLA/IM/2B1—10 wt.% of impact modifier and 2 wt.% of biochar B1; PLA/IM/5B1—10 wt.% of impact modifier and 5 wt.% of biochar B1; PLA/IM/2B2—10 wt.% of impact modifier and 2 wt.% of biochar B2; PLA/IM/5B2—10 wt.% of impact modifier and 5 wt.% of biochar B2.

**Table 6:** Vicat softening temperatures of as-produced and aged PLA samples injected into the moulds with different annealing conditions.

Sample	As-Produced		Aged	
	40 °C	100 °C	40 °C	100 °C
PLA	60.2	60.8	62.4	63.8
PLA/IM	59.4	90.7	62.7	64.1
PLA/2B1	61.2	95.7	63.4	95.5
PLA/5B1	61.2	98.5	68.7	98.5
PLA/2B2	60.2	66.3	62.1	86.4
PLA/5B2	60.2	87.3	63.5	89.7
PLA/IM/2B1	60.4	85.2	61.4	80.5
PLA/IM/5B1	60.3	94.6	61.2	91.1
PLA/IM/2B2	59.7	62.2	61.1	81.4
PLA/IM/5B2	60.0	61.6	62.2	63.4

PLA—neat poly(lactic acid); PLA/IM—10 wt.% of impact modifier, PLA/2B1—2 wt.% of biochar B1; PLA/5B1—5 wt.% of biochar B1; PLA/2B2—2 wt.% of biochar B2; PLA/5B2—5 wt.% of biochar B2, PLA/IM/2B1—10 wt.% of impact modifier and 2 wt.% of biochar B1; PLA/IM/5B1—10 wt.% of impact modifier and 5 wt.% of biochar B1; PLA/IM/2B2—10 wt.% of impact modifier and 2 wt.% of biochar B2; PLA/IM/5B2—10 wt.% of impact modifier and 5 wt.% of biochar B2.

## Data Availability

The data presented in this study are available on request from the corresponding author.
